# Exoskeletons for the rehabilitation of temporomandibular disorders: a comprehensive review

**DOI:** 10.3389/frobt.2025.1492275

**Published:** 2025-05-02

**Authors:** Paul-Otto Müller, Robert Sader, Oskar von Stryk

**Affiliations:** ^1^ Simulations, Systems Optimization and Robotics Group, Department of Computer Science, Technical University of Darmstadt, Darmstadt, Germany; ^2^ Frankfurt Orofacial Regenerative Medicine (FORM) Lab, Department of Oral, Cranio-Maxillofacial and Facial Plastic Surgery, Medical Center of the Goethe University Frankfurt, Frankfurt am Main, Germany

**Keywords:** exoskeletons, robotics, temporomandibular disorders, TMD, rehabilitation, physical therapy, review

## Abstract

Despite the many technological advancements in exoskeletons for the rehabilitation of lower or upper limbs, there has been limited exploration of their application in treating temporomandibular disorders, a set of musculoskeletal and neuromuscular conditions affecting the masticatory system. By collecting data, implementing assisting and resisting training routines, and encouraging active patient engagement, exoskeletons could provide controlled and individualized exercise with flexibility in time and location to aid in the recovery or improvement of jaw mobility and function. Thus, they might offer a valuable alternative or complement to conservative physiotherapy. In this context, the review aims to draw attention to rehabilitating temporomandibular disorders with the help of exoskeletons by looking at the advantages and opportunities these devices potentially provide. After stating the requirements and resulting scientific challenges in various fields and discussing the state of the art, existing research gaps and deficiencies will be discussed, highlighting areas where further research and development is needed.

## 1 Introduction

**FIGURE 1:**
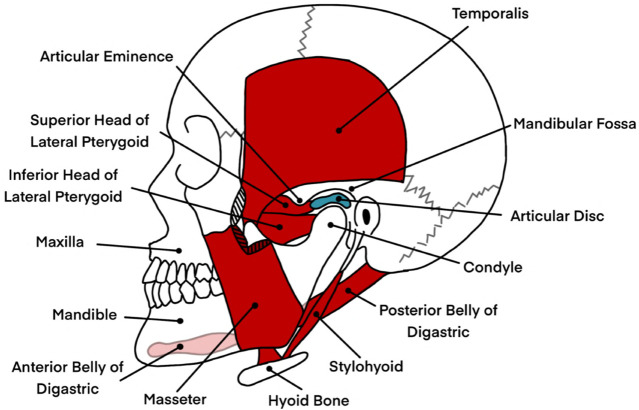
An illustration of the jaw anatomy, highlighting key structures involved in jaw motion and their relevance to the masticatory system. The image displays bony structures in white, including the mandible (lower jaw), maxilla (upper jaw), and hyoid bone, which provide the structural framework for the masticatory system. In red, a selection of muscles responsible for opening and closing the jaw, such as the digastric, masseter, and parts of the pterygoid muscles, are shown. These muscles play critical roles in enabling complex jaw movements such as elevation, depression, protrusion, and lateral excursion. The articular disc, depicted in blue, is positioned between the mandibular condyle and the mandibular fossa of the temporal bone. This disc facilitates smooth articulation by absorbing shear and compressive forces during jaw motion. The illustration excludes ligaments and other soft tissues, such as the temporomandibular joint capsule, which also contribute to joint stability and motion control. This depiction serves as a simplified anatomical reference for understanding the mechanical and biomechanical interactions involved in jaw rehabilitation, particularly in the context of temporomandibular disorders. Information from Okeson was taken as a reference to create the image (Okeson, 2019).

The temporomandibular joints (TMJs) are a unique coupled pair of joints and one of the most complex musculoskeletal systems of the human body, building the operational foundation of the masticatory system. The masticatory system itself, which is involved in tasks such as speech and mastication, consists of two TMJs, which connect the condyles of the mandible or jawbone to the mandibular fossa and articular eminence of the skull’s temporal bone (Wilkie and Al-Ani, 2022; Sagl et al., 2019b). The viscoelastic temporomandibular disc is placed right between the articular surfaces, allowing for separate translational and rotational jaw motions with six degrees of freedom while absorbing applied shear and compressive forces simultaneously. The whole joint, including the disc, is enveloped by a collagenous capsule, sealing the TMJ space. Various ligaments connecting the bony structures support the TMJs, and more than twenty muscles are responsible for generating the appropriate motion. So are the masseter, temporalis, and medial pterygoid muscles activated to open the jaw, while the digastric muscles are mainly responsible for a closing movement (García et al., 2021; Xie, 2016; Sagl et al., 2019b). However, a particular muscle often has multiple functions and can be involved in elevation, depression, protrusion, retraction, and side-to-side motions. The complex kinematics of the mandible are characterized by a pure rotation during the first phase of mouth opening, followed by a combined rotation and translation on a curved path in the second phase (Wen et al., 2015). An illustration of the jaw anatomy is shown in Figure 1. For more detailed information on the masticatory system and its biomechanics, the reader is referred to (Okeson, 2019; Hylander, 2006; Xu and Bronlund, 2010).

Due to the complexity of the masticatory system and the high number of involved structures, it is prone to all kinds of diseases. The term temporomandibular disorder (TMD) is thereby used to describe a wide range of musculoskeletal and neuromuscular conditions affecting the TMJs, the masticatory muscles, and the nervous system controlling them, as well as all associated soft and hard tissues (Durham et al., 2015; Valesan et al., 2021). TMDs are often characterized by orofacial pain, impaired jaw movement, headaches, and/or noises originating from the TMJs (Banerjee et al., 2022; Minervini et al., 2023; Tran et al., 2022). Most symptoms are not directly life-threatening in any form. Still, they can be detrimental in everyday life, as they might impede food intake and speech and worsen psychological conditions like depression or anxiety. Consequently, TMDs might additionally affect patients on a social and emotional level (Vaira and De Riu, 2023).

Recent studies and literature reviews identified a prevalence of 31% for adults and 11% for adolescents and children (Valesan et al., 2021). However, numbers vary significantly in the literature. So concluded Minervini et al. that the prevalence lies between 20% and 60% for children and adolescents, with a higher average number for females (Minervini et al., 2023). Generally, a prevalence of about 10% is cited in the literature (Tran et al., 2022; List and Jensen, 2017; Durham et al., 2015), and presumably, only a portion of the affected have significant symptoms and must thus be treated (Okeson, 2019). This uncertainty in prevalence further reflects the broad range of ailments associated with TMDs and the ambiguity in the diagnosis, which also shows through the differences in classification and non-standardized diagnosis between studies. The diagnosis of TMDs is further aggravated since causes cannot just be physical but can also include a biopsychosocial component so that psychological states might not only worsen due to TMDs but can additionally be a trigger for these kinds of disorders (Li and Leung, 2021; List and Jensen, 2017).

In some cases, the human body might be able to heal and regenerate from TMDs itself without further ado. Still, otherwise, it has been found that TMDs might not be resolved spontaneously in general and might even persist for years (Evans et al., 2016; Li and Leung, 2021). As a result, proper treatment should be sought and applied immediately to avoid developing chronic conditions, which are harder to manage due to physical and mental deterioration (Okeson, 2019; Durham et al., 2015; Li and Leung, 2021). As broad as the range of TMDs so diverse are the rehabilitation and treatment methods developed and put to the test in the last couple of decades. They can be categorized into self-management, conservative, prosthodontic treatment, orthodontic treatment, pharmacological therapy, and surgical approaches. In most cases, reversible non-invasive self-management and conservative strategies are recommended initially, as it has been found that they are often sufficient and can even alleviate symptoms and slow down arthrogenous forms of TMD. Surgical interventions, however, might be inevitable in end-stage arthrogenous TMDs, such as severe osteoarthritis or neoplasms (Durham et al., 2015; Li and Leung, 2021; Medlicott and Harris, 2006; Tran et al., 2022).

Physical therapy in the form of training exercises is a category of conservative methods with supporting literature and data on the efficiency of the rehabilitation of the masticatory system (Armijo-Olivo et al., 2016; González-Sánchez et al., 2023; Tuncer et al., 2013; Tran et al., 2022; Shimada et al., 2023; Herrera-Valencia et al., 2020; Medlicott and Harris, 2006; Nicolakis et al., 2000; 2001; 2002; Xie, 2016). Regularly training the jaw through simple exercises can strengthen jaw muscles, reduce inflammation, and restore the normal motor function of the masticatory system, also after surgeries (Vaira and De Riu, 2023; Gil-Martinez et al., 2018).

Similar to rehabilitative exoskeletons for locomotion or upper limbs like the hands or wrists, exoskeletons for the jaw might prove effective in physically treating TMDs (Plaza et al., 2023; du Plessis et al., 2021). Compared to traditional manual exercise or mechanical and robotic devices, which are either limited in training complexity or availability or are too expensive or heavy, exoskeletons may have unique features and advantages if implemented appropriately. A few of them might be the portability, the consistency of the training as no therapist has to be available continuously, the possible complexity of training routines also aimed at neurological rehabilitation, the tracking of biomechanical, physiological, and training progress metrics, and the controlled and automatically supervised execution of training routines (Evans et al., 2016; du Plessis et al., 2021; Xie, 2016). However, as the development of such devices is in an early stage, there are a lot of open questions to answer, and the scientific foundation to build on still has to be created.

In this context, this review aims to draw attention to rehabilitating TMDs with the help of exoskeletons by looking at the advantages and opportunities these devices might provide. As a result, requirements and scientific challenges in various fields, such as medicine, mechanics, biomechanics, actuation, sensors, and control, will be discussed. In light of the challenges, the current state of the art will be presented while considering biomechanical and mechatronic aspects, highlighting existing research gaps and deficiencies.

## 2 Literature search method

To obtain an overview of the current state of the art of exoskeletons as a rehabilitation approach for TMDs and to fulfill the objectives stated in the introduction, a literature search was conducted in July 2024. Six different databases were searched, including *PubMed*, *CENTRAL* (*Cochrane Central Register of Controlled Trials*), *Springer*, *IEEE Xplore*, and the grey literature databases *Google Scholar* and *WorldCat*. A time frame from 1970 to 2024 was chosen, and the search term comprised the following keywords: [(temporomandibular joint disorders) OR (temporomandibular joint dysfunctions) OR (temporomandibular joint diseases) OR TMD OR jaw] AND (exoskeleton OR orthosis).

A total of 4,275 results were obtained of which 3,729 entries were left after removing duplicates. The duplicates were identified and removed by combining all found entries of all databases and using the *JabRef* bibliography management tool, which searches for similarities in the entries’ authors, titles, and journals, among others (Kopp et al., 2023). Title and abstract screening reduced the number to 23, and six publications were eventually included in the literature review after full reading and applying the following criteria.

**FIGURE 2:**
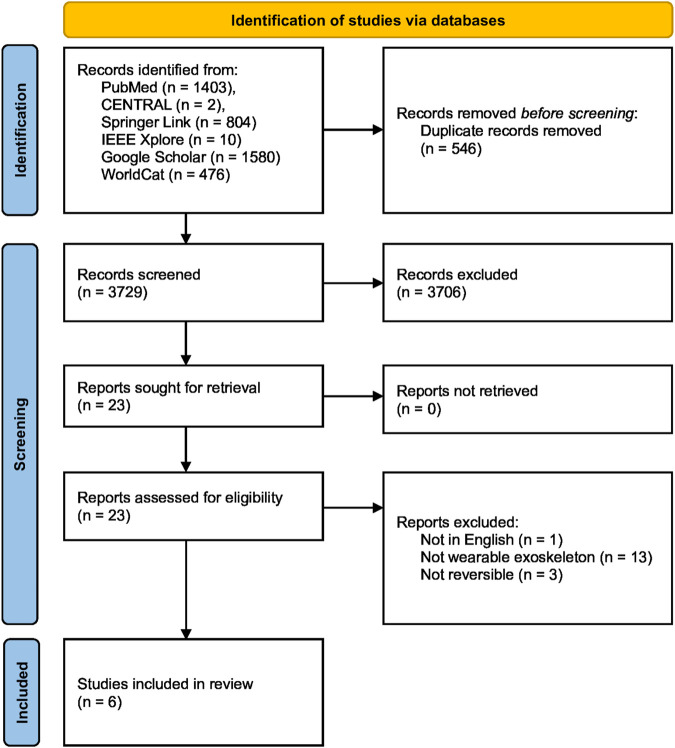
PRISMA flow chart of the literature search and study selection process (Page et al., 2021). The literature search, conducted in July 2024, included six different databases, two of which were grey literature databases, resulting in a total of 4,275 records. After removing 546 duplicates by using the *JabRef* bibliography management tool (Kopp et al., 2023), 3,729 unique records were screened for relevance based on their titles and abstracts. Of these, 23 full-text articles were assessed for eligibility, with 17 articles excluded for various reasons, including the unavailability of an English text, lack of wearability and reversibility in the rehabilitation method, and invasiveness. Ultimately, six publications were deemed eligible and included in the final literature review.

Only texts that were accessible as complete documents and composed in the English language were incorporated. Furthermore, the content had to be about exoskeletons in any form, which implies the wearability and reversibility of the rehabilitation method. Strict exclusion criteria were the invasiveness of an approach. A summary of the literature selection process is shown in Figure 2.

## 3 Temporomandibular disorders

A biopsychosocial model is widely accepted and recommended for diagnosis to describe the multifactorial and complex etiology of TMDs. Beyond physical or biological triggers such as microtrauma, anatomical anomalies, parafunction, or hormonal imbalances, psychosocial factors influencing muscle activity like depression, social stress, or anxieties must be considered as well (Durham et al., 2015; Okeson, 2019; Schiffman and Ohrbach, 2016; Li and Leung, 2021; González-Sánchez et al., 2023). Psychosocial factors can not only influence the predisposition of a person to TMDs but also precipitate or prolong them, leading to chronic pain (List and Jensen, 2017). Special cases to mention include issues arising from tumorous cells, preceding macrotraumatic injuries such as fractures or joint dislocations from incidents like motor vehicle accidents, and sometimes even subsequent surgeries initially intended to improve functionality or enhance aesthetics (Landes et al., 2014; Kretschmer et al., 2019; Khayamzadeh et al., 2022). TMDs can sometimes develop following such macrotrauma or the resulting surgeries.

Furthermore, as it is solely a dental issue and extensively discussed in the literature, malocclusions as possible etiological risk factors are mentioned separately and in more detail. Malocclusions can be caused by either dental or skeletal malformations, leading to impaired static or dynamic occlusion. Dental malocclusion alone, however, is not a risk factor for TMDs unless orthopedic instability combined with significant loading forces is present or the occlusal pattern is altered suddenly or acutely, for example, due to dental crowns. Orthopedic stability means that both the occlusion of the teeth and the musculoskeletal conditions inside the TMJs are simultaneously in a stable state. As a result, applied forces lead to no overloading and injuries of tissues. Sudden changes in the occlusal condition can further influence muscle activity and normal masticatory function, although an adaption of a patient to new circumstances is possible (Okeson, 2019).

Therefore, taking the whole spectrum of the pain’s origin and all etiological factors into account builds the foundation and prerequisite of designing an individualized treatment approach adjusted to the patient’s needs. At first, a standardized diagnosis approach can be followed, but eventually, the actual individual cause must be found if possible.

According to the *Diagnostic Criteria for Temporomandibular Disorders* by Schiffman et al., TMDs can be categorized into four classes depending on their musculoskeletal origin, namely arthrogenous disorders, myogenous disorders, headaches attributed to TMDs, and associated structures (Schiffman and Ohrbach, 2016). Arthrogenous disorders thereby affect the TMJ, ranging from conditions like arthritis and disc displacements to degenerative joint diseases like osteoarthritis to joint fractures and congenital disorders. Myogenous disorders affect, on the other hand, the masticatory muscles, which show through muscle pain, movement disorders, or more severe neoplasms, among others. The primary TMD diagnosis is myalgia, representing approximately 80% of the patients and featuring muscle pain. Myalgia often accompanies arthralgia, a joint-related pain. Disc displacements with and without reduction, impacting about 10% of adolescents and 30% of adults, follow in prevalence based on clinical studies. Comparatively, few cases are diagnosed with degenerative joint disease (Durham et al., 2015; List and Jensen, 2017).

For most of the manifestations of TMDs, at least one treatment method has been developed, even though the effectiveness of one or the other approach might be heavily debated among experts in the field. At least, it is commonly agreed upon that reversible conservative approaches should be used as primary management unless the patient does not respond to them for at least a few months, and irreversible methods are the only viable option (List and Jensen, 2017; Durham et al., 2015; Li and Leung, 2021; Tran et al., 2022). Partially responsible for this paradigm is the fact that most patients, 75%–90% (Durham et al., 2015), will show a positive response to conservative interventions, which bear much fewer risks than, for instance, invasive techniques and might be much more cost-effective. Conservative treatment methods rely thereby on reversible, non-invasive strategies, including occlusal splints and physical, psychological, and pharmacological therapies.

For a more in-depth and complete view of TMDs, their etiological factors, categories, symptoms, and treatment approaches, the reader is referred to (Okeson, 2019).

### 3.1 Physiotherapy

Since complementing, supporting, or even replacing physiotherapy can be the main applications for jaw exoskeletons, it is essential to understand its role in treating TMDs. The efficacy of physiotherapy, a representative within the realm of reversible conservative treatments, has been thereby well-established both independently and as part of a comprehensive multimodal rehabilitation approach for alleviating and resolving symptoms and causes associated with TMDs in both short- and long-term perspectives (Armijo-Olivo et al., 2016; González-Sánchez et al., 2023; Okeson, 2019; Tuncer et al., 2013; Tran et al., 2022; Shimada et al., 2023; Herrera-Valencia et al., 2020; Medlicott and Harris, 2006; Nicolakis et al., 2000; 2001; 2002; Xie, 2016).

Physiotherapeutic measures encompass a spectrum of interventions, including manual therapy involving joint manipulation and muscle mobilization, massage or electrotherapy techniques, and therapeutic exercises such as coordination training, muscle strengthening, and passive stretching (González-Sánchez et al., 2023; List and Jensen, 2017). Recognizing the importance of regular movement in maintaining the health and functionality of synovial joints like the TMJ is a fundamental principle in most of these approaches (Kraaijenga et al., 2014; Israel and Syrop, 1997).

Particularly for myogenous and non-degenerative arthrogenous TMDs, active and passive exercises represent effective, low-risk interventions (Brochado et al., 2018). Exercise-based strategies not only address physical symptoms but may also contribute to reducing patient stress levels and overcoming any apprehension associated with natural jaw movement (Shimada et al., 2023; List and Jensen, 2017).

The combination of manual therapy with physical exercises, or even passive exercises alone, has demonstrated the potential to reverse degenerative changes, alleviate pain, and mitigate motion limitations associated with arthrogenous TMDs, notably disc displacements without reduction (Armijo-Olivo et al., 2016; Wänman et al., 2016; Nicolakis et al., 2001; Israel and Syrop, 1997). Consequently, since myogenous conditions are sometimes accompanied by degenerative changes and joint derangements are generally considered precursors to degenerative joint diseases, timely intervention through physiotherapy might prevent or impede the progression of more severe conditions, such as osteoarthritis (Li and Leung, 2021; Kraaijenga et al., 2014).

In scenarios where conservative strategies yield no response, and invasive intervention becomes inevitable, post-surgical physiotherapeutic measures can serve as a valuable treatment adjunct, contributing to an enhanced overall rehabilitation process and preventing stiffness of the TMJs and tissues (Abboud et al., 2018; du Plessis et al., 2021).

### 3.2 Mechanical and robotic rehabilitation devices for TMDs

In recent decades, various mechanical and robotic devices have emerged to facilitate patient self-training or assist therapists in treatments while aiming to overcome the limitations inherent in conventional rehabilitation services. Such limitations include challenges in accessing physiotherapists in terms of time and location, the subjectivity of evaluating a patient’s condition and subjective non-standardized treatment methods, issues of patient motivation, and the constrained complexity of rehabilitation treatments (Xie, 2016).

As a moderately effective assisting tool and low-cost alternative to therapeutic sessions conducted by a professional, the hand-held unpowered *TheraBite* was developed to be used by the patient on its own at home and treat myogenous TMDs. Operating on a lever mechanism, pressure on the upper and lower teeth can be applied to support opening the jaw, exert resistance on closing, and stretch the muscles and other soft tissues (Kraaijenga et al., 2014; Heres Diddens et al., 2016). However, its unidirectional operation, potential tooth damage, and reliance on user motivation and endurance pose limitations. Moreover, appropriate execution of the training is no longer guaranteed, raising concerns about proper execution and potential further harm to mandibular structures. Similar devices with comparable functionalities and similar limitations can be found in the literature (Lo et al., 2008; Sun et al., 2015).

Moving towards more advanced robotic devices, Takanobu and Okino et al. developed the *WY* (*Waseda-Yamanashi*) robot series around 2000 (Takanobu et al., 1999b; a, 2000a; b, Takanobu et al., 2002; Takanobu et al., 2003; Okino et al., 2004). Employing a master-slave concept, a therapist operated the robot, which utilized a six degrees of freedom (DoF) parallel mechanism to replicate the masticatory system’s complete range of motion (RoM). The upper jaw was placed on an upper mouthpiece, while the lower jaw was fixed between a lower mouthpiece and a chin holder, enabling the parallel system to move the mandible. Several sensors were incorporated to monitor the training process and implement safety measures, such as electromyography (EMG), axial force, and biting force sensors. However, its considerable size, complex structure, weight, and cost make it less practical for common physiotherapeutic use. Additionally, its operation requires an expert’s intervention.

As massage techniques might be useful as a treatment modality, especially in the early stages of TMDs, robotic systems for this task have been investigated. Among them is the *WAO* (*Waseda-Asahi Oral-Rehabilitation*) robot series, comprising two independent six DoF arms with interchangeable plungers, performing pressing or rubbing movements to stimulate facial tissues physically. Safety measures were implemented by bounding the rotational speed and positions and limiting motor currents and pressures applied to the patient’s face. An admittance controller for the plunges’ positions further introduced virtual compliance to the system, enabled by a 6-axis force sensor at the end-effector. Utilizing a human head model based on computed tomography or magnetic resonance imaging data while considering the elasticity of soft tissues, a trajectory for the plunges could be calculated (Koga et al., 2007; Ariji et al., 2009; Ishii et al., 2009b; a; Solis et al., 2009; Hiraiwa et al., 2013; Ariji et al., 2015; 2016). While subjected to similar restrictions as the *WY* robots and lacking a long-term effectiveness study, it may serve as a supplementary treatment option, potentially relieving therapists.

Another more recent approach to rehabilitation robotics was presented by Kalani et al. involving a Gough-Stewart platform, where the jaw rests on a mobile plate connected to a stationary base by six linear actuators (Kalani et al., 2016; 2018; 2019). However, the details regarding the connection between the platform and the mandible remain unclear. Notably, the research prioritizes generating plausible, natural trajectories for potential rehabilitation robots over the robot’s construction, suggesting that this concept requires further development.

In summary, while current physiotherapeutic devices demonstrate conditional effectiveness in addressing various TMDs, they are constrained by limited functionality, practicality, and availability. Additionally, these devices only partially address some of the previously mentioned constraints associated with conventional therapies conducted by a physiotherapist. Consequently, there is a need for more advanced devices that can accurately capture the intricate kinematics of the masticatory system while maintaining practicality, combining the advantages of straightforward mechanical devices with the complexity inherent in robotic systems. Notably, one promising direction in this pursuit is the exploration of jaw exoskeletons, which will be further discussed in the following sections.

## 4 Related state of research of exoskeletons for rehabilitation

Rehabilitative exoskeletons are wearable robotic devices designed to assist, enhance, or restore the physical capabilities of individuals undergoing rehabilitation. These exoskeletons typically comprise a combination of mechanical structures, sensors, and actuators that work in tandem with the user’s body to facilitate controlled movements and support the rehabilitation process by assisting or resisting the patients during training. Research regarding exoskeletons for lower or upper limb rehabilitation has been ongoing for a couple of decades and has seen significant advancements in the last years (Bao et al., 2019). These devices’ actuation, sensing, and mechanical design aspects have been extensively studied and developed, and various control strategies have been proposed to address the challenges associated with assisting and training during rehabilitation, e.g., (Tucker et al., 2015). While the transition to jaw exoskeletons involves unique challenges due to the smaller size, intricate anatomy, and specific functional requirements of the masticatory system, insights from limb exoskeletons can inform jaw exoskeleton development in regards to sensor systems (Novak and Riener, 2015), actuator concepts (du Plessis et al., 2021; Plaza et al., 2023; Tiboni et al., 2022), or control strategies (Tucker et al., 2015; Dalla Gasperina et al., 2021; Maggioni et al., 2018).

Unlike limb exoskeletons, neck exoskeletons share a closer anatomical and functional similarity to jaw exoskeletons due to their proximity to the masticatory system and their focus on supporting head and neck movements. Insights from neck exoskeleton designs, such as lightweight actuation and head-mounted systems, might provide a more direct reference for jaw exoskeleton development. For example, the active device by Demaree et al. consists of three parallel linkage systems attached to the shoulders and the head, supporting neck and head movements for patients with amyotrophic lateral sclerosis. The mechanical structure was improved by employing an optimization scheme maximizing the range of rotation and transmission efficacy (Demaree and Zhang, 2023; Demaree et al., 2024). Cho et al. developed a neck exoskeleton to mitigate muscle fatigue during prolonged neck flexion by connecting a head support to a neck brace and vest with cables controlled by a clutch (Cho et al., 2024). Garosi et al. designed a passive neck exoskeleton to alleviate overhead work-related neck strain by employing an U-shaped headrest with an adaptive jack connected to the back and hips (Garosi et al., 2022). All three devices included user studies demonstrating their effectiveness in supporting the intended motions. One insight that can be drawn from these neck exoskeletons might be to reduce the load on the head and neck by distributing it to other body parts by placing the actuators and processing units on the shoulders or back.

In conclusion, the development of a jaw exoskeleton for TMDs, as discussed in the next section, can leverage advancements made in the field of existing, more advanced rehabilitation devices. However, additional efforts are required to address the unique challenges associated with jaw exoskeletons, including the complex anatomy and dynamics of the masticatory system, the smaller scale, and the difficulties in securely attaching it to the head and jaw.

## 5 Exoskeletons as a promising treatment approach for TMDs

The execution of jaw motions involves a feed-forward component for pre-programmed movements, a periodic motion component produced by the central pattern generator, and a voluntary sensor-supported component for overcoming food resistances or adapting to different food textures (Xu and Bronlund, 2010). The rhythmic nature, similar to bipedal locomotion, the proven effectiveness of exoskeletons for human limb rehabilitation, and the similarity of the masticatory system to other parts of the human body make an exoskeleton seem a suitable and promising rehabilitation approach for TMDs (Takakusaki, 2013; Rehmat et al., 2018). In the context of TMDs or jaw-related rehabilitation, a jaw exoskeleton would aim to provide targeted support and controlled motion to aid in the recovery or improvement of jaw mobility and function, improving quality of life.

### 5.1 Potential advantages and opportunities

Exoskeletons, functioning as assisting physiotherapeutic devices, offer several potential benefits compared to traditional methods. While they may not independently address all types of TMDs, they present a valuable alternative or complement to conservative physiotherapy, potentially proving effective within a multimodal treatment strategy as outlined in this section.

#### 5.1.1 Reducing the limitations of a therapist

Given that the considered jaw exoskeletons fall under the category of non-invasive physiotherapeutic approaches, they should encompass the benefits of traditional physiotherapy and, presumably, exhibit similar effectiveness as traditional physical exercises. What might set them apart in certain aspects is a robotic system’s inherent precision, repeatability, complexity, and ability to operate autonomously and document its motions. Consequently, such systems are not dependent on the physiotherapist’s skills and endurance, potentially providing more intensive and consistent rehabilitation sessions. Due to the autonomy and portability, a therapist may not be needed at all during the training sessions, making the choice of the training location more flexible for the patient and relieving therapists simultaneously (du Plessis et al., 2021; Xie, 2016). Thus eliminating the challenges of accessing a therapist regarding time and location and making efficient treatment feasible even for individuals with time constraints or other disabilities. However, initial usage instructions and regular update meetings with the therapist remain relevant.

#### 5.1.2 Training modalities

Moreover, exoskeletons can make it possible to carry out predefined training routines in a controlled and autonomously supervised manner, accommodating various training modalities. These modalities include resistance training, which opposes the user’s jaw motion, thereby strengthening masticatory muscles. Passive stretching or manipulation of the mandible can activate joint metabolism and improve the RoM. Additionally, the assist-as-needed paradigm can be employed, providing support only when necessary to achieve specific training goals promoting active participation and preventing overreliance on the device. This paradigm can be implemented through adaptive controllers that continuously monitor patient effort and engagement, gradually reducing assistance as performance improves. For jaw exoskeletons specifically, this might involve force sensors detecting patient-initiated movements and the system providing complementary forces proportional to the detected performance gap. Finally, constraint-induced strategies involve restricting mandibular movement in specific directions, preventing compensatory motions, and ensuring proper movement patterns (Iandolo et al., 2019; Dalla Gasperina et al., 2021).

#### 5.1.3 Adaptability

Contrary to existing mechanical and end-effector-based robotic devices, advanced jaw exoskeletons might be able to capture the complex intricate kinematics of the masticatory system, mapping all six DoFs while maintaining flexibility and practicality. This property is achieved through direct contact with the patient’s masticatory system and by leveraging techniques from other exoskeletons, involving a thoughtful choice of actuation, power transmission, and mechanical design (Plaza et al., 2023; du Plessis et al., 2021). Similarly, an adaption to the patients’ differences and various kinds of TMDs might be possible.

#### 5.1.4 Activity and progress tracking and data collection

An exoskeleton typically incorporates various sensors. These sensors can now not only be used to control the system but also to collect data that reflects the patient’s current condition, making it possible to optimally adapt the training correspondingly and to change the difficulty of the training (Xie, 2016; Yip et al., 2023). Metrics such as muscle activities, exerted forces, and accelerations can be used to personalize training and track progress by comparing values with those of healthy subjects. Additionally, comparisons between patients and cases of TMD are possible. Significantly, this data may contribute to more objective diagnosis and prognosis, potentially leading to treatment standardization.

#### 5.1.5 Neurological and neuromuscular rehabilitation and feedback

In neurological and neuromuscular rehabilitation, particularly for enhancing the re-learning of motor skills, providing appropriate feedback and progressively challenging tasks across the entire range of possible movements is crucial. Neurological and neuromuscular rehabilitation addresses neurological or neuromuscular issues affecting the nerves controlling the masticatory muscles.

For effective neuromuscular rehabilitation, jaw exoskeletons can employ targeted resistance patterns that adapt to the specific muscle groups requiring strengthening. By modulating assistance levels during different phases of jaw movement, these devices can selectively engage weak muscle groups while supporting compensatory patterns that may have developed due to pain or dysfunction. Biofeedback mechanisms using EMG signals can help patients visualize muscle recruitment patterns, facilitating conscious motor control retraining and muscle re-education. Feedback mechanisms can also include previously recorded trajectories with position, velocity, or force information (Levin and Demers, 2020; Huang and Krakauer, 2009; Iandolo et al., 2019). Additionally, a game-like assist-as-needed setup with visual feedback can encourage the active participation of the patient, potentially enhancing the effectiveness of the motor learning process (Evans et al., 2016; du Plessis et al., 2021; Levin and Demers, 2020).

Virtual reality as a form of visual feedback has found widespread use in the rehabilitation literature in recent years, supporting or enhancing the treatment process for patients with, for instance, chronic strokes or Parkinson’s disease (Gonzalez-Argote, 2022; Laver et al., 2017). By using visual perturbations leading to compensatory motions, virtual reality can be used for balance and stability training (Chander et al., 2019; Ketterer et al., 2022). Like a mirror, virtual reality could let a TMD patient consciously perceive their jaw motions. Whereby optical perturbations might help reduce evasive movements acquired due to the patient’s previous pain gradually. By showing a jaw position different from the actual one, the patient might try to correct the movement according to the visually perceived one. A high enough degree of immersion into the virtual environment is mandatory (Caserman et al., 2016).

To realize these potential benefits, numerous scientific challenges must be overcome across various fields, and essential biomechanical and mechatronic requirements must be met. This complexity suggests the need to prioritize specific functionalities depending on the intended goals and the type of TMD to be treated, keeping the complexity and, thus, research and development efforts within limits.

### 5.2 Requirements and scientific challenges

By examining the intended purpose and expectations of a rehabilitative jaw exoskeleton alongside the biomechanics of the masticatory system, essential general and jaw-specific requirements and the resulting scientific challenges can be derived.

#### 5.2.1 User safety

First and foremost, ensuring user safety during physical interaction is paramount in designing a rehabilitative jaw exoskeleton. The challenge begins with power transfer, as the device applies forces to the mandible during training and responds to varying forces exerted by the patient. Establishing a reliable power transfer necessitates a robust mechanical structure capable of applying forces to the jaw, a challenge given the unique anatomy of the masticatory system that limits force application points to the region around the mouth and chin. Simultaneously, the control concept must adapt to changes in applied accelerations and variations in biomechanical parameters due to the tensions of muscles and other soft tissues while meeting safety constraints and providing optimal assistance.

Kinematic compatibility is another crucial aspect of safety, requiring the device to adhere to the natural anatomy of the masticatory system, move in human-feasible ways to avoid harm to the jaw, and adapt to changes in biomechanical parameters (Pons, 2010). Passive or active compliance, achieved through hardware or software techniques like springs or impedance controllers, is often introduced into a robotic system to help simplify kinematic compatibility and cope with unexpected behavior. More recent research emphasizes soft or hybrid designs to partially mitigate the disadvantages of rigid exoskeletons (Morris et al., 2023). For the jaw, a soft mechanism approach, leveraging innate compliance and distributing forces on the facial area with a mask-like design, may relieve stress on the chin and teeth, enabling robust power transmission and compatibility with natural motions. However, this approach must consider challenges such as material choice, actuation, compliance with pressure limits, and complex control due to a high number of DoFs.

Contributing further to the system’s safety is a reliable sensor concept, resilient against sensor drift and placement, external influences, and user variability (Yip et al., 2023). Various sensors, including inertial measurement units (IMUs), gyroscopes, angle sensors, position sensors, and force sensors, must be integrated to measure the device and user’s current state redundantly, enhancing controllability and detecting potentially dangerous system states. A powerful processor or microcontroller is also essential to process sensory information in real-time. Fail-safe mechanisms should consistently be implemented as a last resort.

#### 5.2.2 Robust human-robot cognitive interaction

In addition to ensuring a reliable physical interaction between the patient and the exoskeleton, a robust cognitive interaction is indispensable for implementing training routines based on the assist-as-needed paradigm and detecting changes or abnormalities in the user’s behavior (Pons, 2010). This requirement indicates overcoming challenges in incorporating suitable sensory systems. Typically, robotic devices and exoskeletons utilize surface EMG electrodes or electroencephalography (EEG) sensors to detect the user’s intention. EMG electrodes measure electric muscle activity, while EEG sensors attached to the skull provide information about the temporal evolution of the brain’s electric fields. Despite their potential to detect intention, both sensor types are susceptible to noise and are influenced by skin properties such as electrical resistance, hydration level, and hairiness. Notably, EEG signals often exhibit a low signal-to-noise ratio and significant variability between subjects, exacerbated by pathologies, demanding customized use, and robust data processing methods (Yin et al., 2020; Tiboni et al., 2022). Additional force myography and camera-based systems may complement EMG or EEG sensors, and already mentioned sensors such as IMUs could also be applied to detect user motion and intention (van Dellen et al., 2023; Sierotowicz et al., 2022). By fusing data from multiple sensors of the same or different kind and thus mitigating the individual disadvantages, the system can enhance the reliability of user intention detection (Novak and Riener, 2015). Afterward, machine learning algorithms can be used to interpret and classify the data from these sensors (Coser et al., 2024).

Looking at related literature, interaction control strategies often employ a hierarchical structure, as in the proposed generalized control framework for active lower limb devices by Tucker et al. (Tucker et al., 2015). A high-level controller detects and estimates the user’s intention. At the mid-level, this detected intention is translated into the desired motion for the device. Finally, at the lowest level, the device executes the specified motion. However, there are still open questions regarding the robust detection of human intention, the accurate motion control with the given intention, and the optimization of control parameters to accommodate different individuals (Li et al., 2021).

In conclusion, designing robust human-robot interactions and integrating mentioned sensors into the overall system requires a sophisticated algorithm capable of leveraging the provided data, extracting essential user intentions, and addressing individual differences and noise effects.

#### 5.2.3 Forces and range of motion

Requirements for designing the actuator properties and the device’s RoM can be discerned from studies in the literature. So concluded Hansma et al. that an estimated force of approximately 15 N–25 N is necessary to open the jaw without active muscle participation, setting a lower threshold for the applicable force in a jaw exoskeleton (Hansma et al., 2006). Additionally, Brunton et al. identified an average maximal opening force of 79 N for men and 41.16 N for women, providing insights into the forces required for implementing opening resistance training (Brunton et al., 2017). For a general upper boundary, the maximum occlusal force in the incisor region in a closed state of about 100 N may serve as an indicator, even though the jaw is capable of generating well over 500 N in the molar area (Wen et al., 2015; Xu and Bronlund, 2010). Generally, the mouth can be opened vertically in the range of 35 mm–60 mm, with the first 20 mm–25 mm characterized by the pure rotation of the TMJs, followed by translational motion along a curved path (Svechtarov et al., 2015; Wen et al., 2015). In lateral and protrusive directions, forces up to 9.4 N with a displacement of 1.6 cm and up to 13.1 N with a displacement of 1.0 cm were recorded, respectively, acting against passive muscle forces only. Notably, the achievable RoM can surpass what can be achieved by active muscle use alone, and resistance increases closer to the motion limits (Hansma et al., 2006). The minimum dynamic response of the actuators may be determined by the typical chewing frequency, which ranges from 0.95 Hz to 2.17 Hz (Wen et al., 2015).

Overall, the challenge lies in developing an actuator concept that accommodates all possible and needed DoFs to naturally replicate the movements of the masticatory system, engaging all muscles and associated components appropriately in the rehabilitation process.

#### 5.2.4 Wearability and portability

The exoskeleton must embody wearability and portability to fully leverage the potential benefit of freely accessing the training device without a continuous need for a therapist. Accordingly, a design and material that is biocompatible and lightweight while concurrently meeting safety requirements, such as robust power transmission, must be selected. While no specific recommendations on the weight of jaw exoskeletons were found, studies on the wearability and comfort of helmets, headsets, or head-mounted displays may provide valuable insights. For instance, Odell and Dorbala defined “comfortable wear time” as the duration a helmet or headset can be worn without causing moderate discomfort (e.g., fatigue or contact pressure) and the wearer’s willingness to continue wearing it (Odell and Dorbala, 2024). Their study, conducted with 16 participants and headsets weighing between 0.5 kg and 0.6 kg, revealed that for the lower quartile of participants, the average comfortable wear time decreased by 11 min for every 33 g increase in weight. Specifically, participants in this group experienced a reduction in wear time from 71 min at 0.5 kg to 37 min at 0.6 kg. Overall, females appeared to have a shorter comfortable wear time than males. Furthermore, Ito et al. emphasized the importance of considering weight distribution and proposed the torque at the neck joint as a measure of the load induced by a head-mounted display (Ito et al., 2021). Consequently, both weight and balance, as well as gender-specific differences in the development of discomfort, must be considered.

Commonly used actuators in existing powered rehabilitative exoskeletons operate on electromechanical principles, usually combined with gears and cable systems for power transmission. Although less prevalent and predominantly for lower limb devices, pneumatic and hydraulic actuation is employed, with increasing attention in the research literature (du Plessis et al., 2021; Plaza et al., 2023). Each concept has advantages and disadvantages impacting dimensions, weight, compliance, control accuracy, and applicable forces. Electric solutions, being more efficient, accurate, and reliable in control, necessitate additional measures like gears or springs to ensure back-drivability and compliance. Hydraulic systems offer inherent compliance and high loads but are bulky and require a tightly sealed hose system. In contrast, pneumatic actuators feature inert compliance, a high power-to-weight ratio, and lightness. However, they generally lag behind electric and hydraulic systems concerning applicable forces, torques, and bandwidth due to gas compression. Fluidic actuator concepts furthermore require a pressure source, increasing the weight and worsening the portability. However, such as PAMs (pneumatically actuated muscles), these systems might be able to mimic the individual masticatory muscles, even though they work only unidirectionally. To address the individual drawbacks of these solutions, hybrid actuator systems, combining electric with fluidic approaches, have been proposed (Tiboni et al., 2022; Agarwal and Deshpande, 2019; Dittli et al., 2021). Since the applicable load on the head is limited, a high power-to-weight ratio or redirecting the force from an actuator on the hip, for instance, is mandatory.

In the realm of soft, lightweight designs, researchers are exploring additional actuator concepts. Ongoing investigations include soft dielectric elastomer actuators or ionic polymer-metal composites, deforming when electrically charged. The former boasts high bandwidth and performance but requires a relatively high voltage, while the latter functions well with only a few volts but at the cost of reduced power density (Youn et al., 2020; Pan et al., 2021; Yang and Wang, 2024). Beyond that, soft electrical systems can be integrated with fluids, producing dielectric fluid electrostatic actuators. Considering the earlier requirements, these soft actuators, combined with auxetic or transformable materials, forming a mask-like structure capable of bending, twisting, and contracting, hold promise for research in portable jaw exoskeletons (Morris et al., 2023). Regardless of the actuation principle, the power supply must be considered when selecting the components. Whether the device is powered by a battery, a cable, or a hybrid solution, the power supply must be likewise reliable, lightweight, and long-lasting, ensuring continuous operation during training sessions.

#### 5.2.5 Flexibility and data collection

A potential jaw exoskeleton must be flexible enough to accommodate the individual differences of patients in terms of anatomical and biomechanical features and, to some extent, pathologies. Moreover, to enhance the objectivity of diagnosing and prognosing TMDs, monitoring training progress, and facilitating adaptations, the system should effectively gather, process, and export relevant data. This necessitates substantial storage capacity, powerful onboard processors, and a reliable connection to a host computer. The sensors already employed for control and user intention detection can be repurposed for data acquisition.

Multimodal sensing approaches enhance training precision by combining complementary data streams–for example, integrating force sensors to measure jaw pressure, IMUs to track motion trajectories, and EMG sensors to monitor muscle activation patterns. This sensor fusion compensates for individual sensor limitations and provides a more complete picture of the patient’s performance and condition. Recent advances in additive manufacturing techniques allow for direct embedding of sensors within the exoskeleton structure during fabrication. Multi-material 3D-printing can create structures with integrated strain gauges, capacitive sensors, and conductive pathways, reducing bulk while increasing sensor density and conformability to the patient’s anatomy (Liu et al., 2021; Su et al., 2023; Hossain et al., 2024; Mitra et al., 2024; Perilli et al., 2024).

To that end, training adaption, progress tracking, and objective diagnosis might benefit from the integration of machine learning algorithms, drawing conclusions from the provided sensory data, distinguishing between TMDs, and evaluating the patients’ states. Furthermore, a critical aspect involves assessing the efficacy of a jaw exoskeleton across diverse patients with varying TMDs through comprehensive user studies and the analysis of acquired data. This information can be a foundation for developing more individualized and specialized exoskeletons and tailor-made training routines.

#### 5.2.6 Neurological and neuromuscular rehabilitation

After delving into the necessary technical attributes of the exoskeleton, the subsequent considerations shift more toward the rehabilitative and user perspectives. As it is an essential component in neurological and neuromuscular rehabilitation and enhances the treatment effectiveness, the patient must be encouraged and actively involved in the training process. In order to accomplish active participation of the patient, assist-as-needed routines and game-like tasks could be implemented, assuming that the mentioned actuation and RoM requirements are fulfilled. Consequently, graphical user interfaces and algorithms predicting or reacting to the patient’s trajectories must be developed. One graphical task might be to follow a predefined trajectory with the jaw, while another might apply a constant predefined force. For example, the patient’s performance can be evaluated by comparing the actual trajectory with the predefined one.

For neuromuscular rehabilitation specifically, the system must be capable of targeting specific muscle groups requiring strengthening. This requires the ability to modulate assistance levels during different phases of jaw movement to selectively engage weak muscle groups while supporting compensatory patterns that may have developed due to jaw pain or dysfunction. The visualization of EMG-based biofeedback might facilitate conscious motor control retraining and muscle re-education.

Additionally, the patient’s facial expressions and body language can be monitored by camera-based systems to detect signs of discomfort or fatigue, providing feedback to the system and the therapist.

#### 5.2.7 Usability and acceptance

Since the device’s end user is the patient, usability and user acceptance should be considered early on (Pons, 2010). Usability is facilitated and improved through a straightforward installation process and an intuitive exoskeleton operation. In parallel, acceptance is fostered by a design that prioritizes comfort and ergonomics, ensuring adherence to pressure limits and movement constraints while affording the user complete control over the device. For instance, a hand-held button must be continuously pressed to keep the device operational. Release of the button results in a power cut-off, assuming the actuators are backdrivable. Ideally, patient preferences should be incorporated during the initial phases of designing and developing the exoskeleton concept.

#### 5.2.8 Biomechanical model and adaptive control

The last crucial aspect that holds significance for both technical development and medical applications involves establishing a comprehensive biomechanical model of the masticatory system in conjunction with the exoskeleton. Concerning device development, employing a model stands out as the most adequate approach for validating and testing the integrity of the design. A model enables the simulation and evaluation of the exoskeleton’s functionality, identification of potential issues, and ensuring alignment with its intended objectives before committing resources to construct a physical prototype. Specifically, force relations within the joints can be observed when applying the exoskeleton, a challenging task on an actual patient. For the model to be a reliable representation of the masticatory system, real-world data must be acquired for optimization and validation. Such data can include magnetic resonance and computed tomography images or motion capture and bite force information obtained from healthy individuals and patients with TMDs. Optimal control or machine learning techniques such as Reinforcement Learning might additionally help to identify neural and muscle activation patterns that can be used to drive the model during testing of the exoskeleton (Abdi et al., 2020).

From a medical standpoint, the model facilitates comparisons between healthy individuals and those affected by TMDs or between patients with distinct TMD conditions. By integrating previously acquired data, one can discern alterations in natural movements and acquire insights into pathological biomechanics. Furthermore, the model even allows for the representation of the effects of joint disc deformations. An individual differences adaptive model that merges classical multi-body dynamics with finite element methods would be imperative to achieve this. However, this is not yet an established, common approach. In the context of jaw models, notable recent research by Sagl et al. features bones as rigid bodies, muscles, ligaments, and cartilage as spring-like components, and the TMJ capsules and discs as finite element models (Sagl et al., 2019b; a, 2021). Highly detailed and sophisticated models based solely on the finite element method also exist, emphasizing the mechanical properties of the jaw, such as stress or strain relationships, rather than its overall kinematics or dynamics. An example of such research is by Kober et al., who explore, among other things, the effects of altered biomechanics due to diseases or surgeries (Kober et al., 2004; 2015; 2017). For an overview of biomechanical human jaw models, refer to the recent review by De Stefano and Ruggiero (De Stefano and Ruggiero, 2024).

Model-based control strategies are often employed to enhance control accuracy but with a higher computational cost. Adaptive model predictive controllers even use a parameterized model as a basis for control to mitigate prediction errors. To adapt to differences in the patient’s anatomy, applied accelerations, or variations in biomechanical properties, e.g., due to the tensions of muscles and other soft tissues, admittance or impedance controllers can be used, providing a mass-spring-damper-like behavior and adding artificial compliance to the system (Dalla Gasperina et al., 2021; Maggioni et al., 2018). Related surveys describe, e.g., active compliant control in robotic systems (Schumacher et al., 2019) and force-impedance control and their unification (Haddadin and Shahriari, 2024).

By leveraging learning-based estimation algorithms, incorporated sensors can be used to detect the patient’s movements and adapt a previously created template model to the patient’s characteristics (Yip et al., 2023). Besides the model itself, machine learning approaches can be used to optimize the control parameters and individualize the control strategy for each patient (Coser et al., 2024).

### 5.3 State of the art

The literature search revealed that the development of jaw exoskeletons is limited to the research sector, and no commercial or industrial products are currently available. However, looking at the current state of research, several research groups have developed jaw exoskeletons in the last couple of years, each with distinct characteristics and objectives. The following section will overview this field’s most recent and relevant research chronologically. As this research area is still at an early stage of development, the first relevant work dates back to 2010, and only a few concepts have been proposed since then.

#### 5.3.1 A helmet-mounted jaw exoskeleton for rehabilitating temporomandibular disorders

**FIGURE 3:**
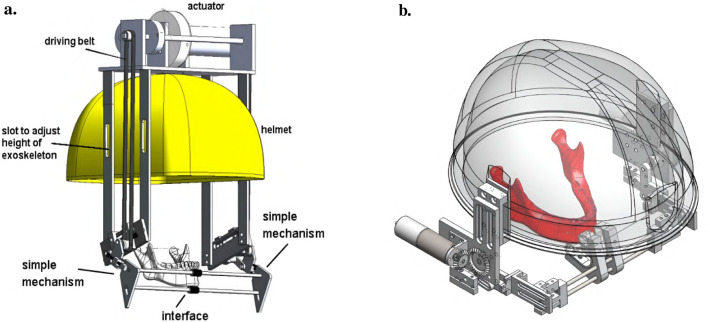
Two versions of the jaw exoskeleton developed by Wang et al. **(a)** The first version comprises a rigid four-bar linkage system attached to a helmet, driven by a DC motor placed on top of the helmet and connected via a belt to the linkage system. The chin is placed in between two cushioned bars to transmit the forces. The links are adjustable to accommodate different patients and trajectories. A model of a human mandible can be seen in between the chin holder bars. ^©^2010 IEEE. Reprinted with permission. **(b)** The second version is similar in design but features a more compact structure. Two DC motors are placed on the sides of the helmet and are connected to the linkage system via gears. The chin holder has a more complex design, and hidden springs are added to introduce passive compliance to the system. A human lower jaw is depicted in red. The material used for the linkage system in both designs is aluminum. Reprinted with permission by the author. **(a)** The first device version by Wang (2010). **(b)** The second device version Wang (2014).

The first concept of a jaw exoskeleton that was found in the literature was developed by Wang et al., in 2010–2014 (Wang et al., 2010; 2014; Wang, 2014). Based on two rigid four-bar linkage systems mainly made of aluminum and attached to a helmet, the device was designed as a rehabilitation tool for patients with TMDs, weighing no more than 1,000 g. Since the system was developed by simplifying the mandibular kinematics to the 2D sagittal plane, it can only assist with opening and closing in the vertical direction with one DoF. The linkage system’s dimensions were roughly selected using a graphical simulation tool initially, after which the fine-tuning was done by optimizing the lengths with previously recorded trajectories of the incisor and condylar points of the jaw. Consequently, this system can closely reproduce the mandibular kinematics in the sagittal plane. To generally be able to generate a group of different trajectories, the linkages of the exoskeleton were designed to be adjustable. A chin holder realized the power transmission to the mandible while some compliance was added by integrating physical springs into the system; however, not in the lateral direction. Two versions of the device can be found in the literature, which is depicted in Figure 3.

For the actuation, a DC motor was selected so that the device could output an estimated force range of 10 N–30 N. A manually tuned PID controller was implemented to control the position of the chin attachment. Furthermore, various sensory systems were integrated to measure forces and angles, and Hall sensors were utilized to detect motion limit violations and cut the system off power. The validation of the system was separated into the evaluation of the mechanical design and the human-machine interaction. A stress and strain analysis with 200 N applied to the chin holder yielded a maximal pressure and displacement of 40 MPa and 0.2 mm, respectively, confirming the stability. In addition, a dynamic simulation including a model of the 4-bar linkage system, a gear, and the DC motor provided the means to tune the PID controller.

**FIGURE 4:**
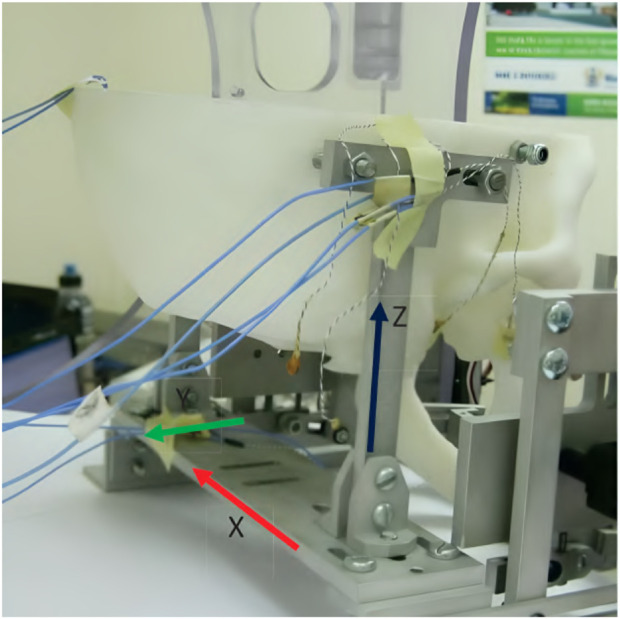
A 3D-printed model of the skull and jaw to evaluate the jaw exoskeleton concept. The skull is fixed to a base plate, and the exoskeleton is attached to the freely moving jaw. Sensors are placed inside the joints measuring forces in a range of 15 N–60 N during the interaction. The condyles and incisor points are tracked by an electromagnetic-based recording device (Wang, 2014). Reprinted with permission by the author.

Regarding the human-machine interaction, both a simulation environment and a physical prototype were created and 3D-printed (Figure 4). In the simulation, the device was operated while interacting with a jaw model, which consisted of the mandible simulated as a rigid geometric model, ligaments modeled as springs, muscles where only the passive parts of Hill-type actuators were considered, and joint discs modeled as a series of springs and dampers. It was concluded that a maximum force of 45 N is present inside the TMJs during the opening of the mouth. Notably, the model of the jaw was never validated with proper data.

The physical model comprised the skull and four pairs of passive muscles split into one ore more muscle cords–temporalis, masseter, pterygoid, and digastric muscles. Force sensors (*FlexiForce*) placed inside the joints recorded a force of 15 N–60 N during interaction with an exoskeleton prototype. Additionally, an electromagnetic-based recording device (*Articulograph AG500*) tracked the condyle and incisor point trajectories. However, the acquired data was only compared to the exoskeleton’s calculated trajectories, not the prototype’s, showing a maximum deviation of 2 mm and many oscillations, explained with motor shakings and unstable fixations. Since the exoskeleton was still in an early development stage, no clinical studies were conducted.

#### 5.3.2 A shoulder-mounted exoskeleton concept for rehabilitating temporomandibular disorders

**FIGURE 5:**
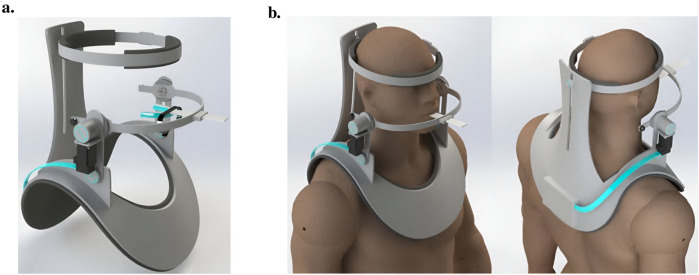
An illustration of the jaw exoskeleton concept by Evans et al. (2016). **(a)** The image shows the device with the shoulder mount, the head brace, and the mouthpiece linked through a linear slide to a bracket attached to an actuator system. ^©^2016 IEEE. Reprinted with permission. **(b)** The device is mounted on the patient’s shoulder and provides assistance in the vertical direction. The mouthpiece can passively compensate for the mandible’s translational motions through a linear slide. A brace fixes the head in place. The processing unit is worn on the back of the device and patient, connected to the motor by a cable shown in turquoise. ^©^2016 IEEE. Reprinted with permission.

The next exoskeleton was developed by Evans et al. a few years later in 2016 (Evans et al., 2016). However, it was just a concept that was neither realized as a physical prototype nor modeled in simulation. The research objective was to develop a portable, practical robotic device especially targeted at neurological rehabilitation and at the adaption to the different manifestations of TMDs by implementing different training routines. A shoulder-mounted rigid, rotating bracket with an attached mouthpiece and a chin strap characterizes this device, allowing assistance in the vertical direction (Figure 5). The main structure should be made of aluminum and heat-formed polyvinyl chloride (PVC) sheets.

**FIGURE 6:**
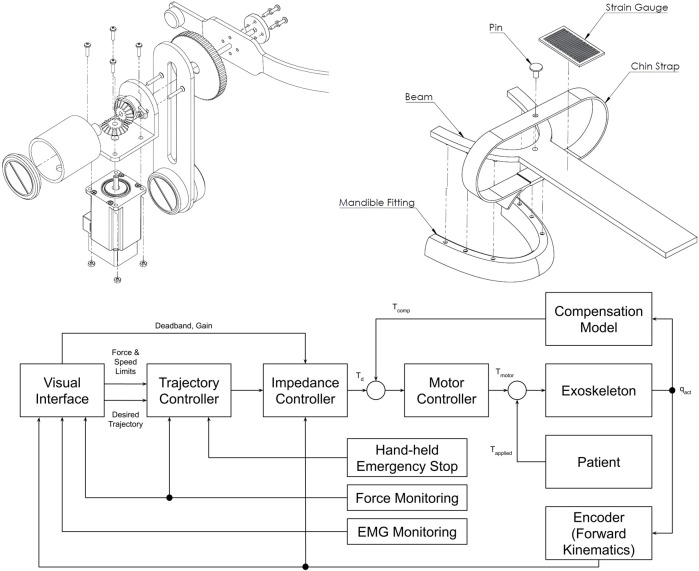
The drive mechanism, mouthpiece, and control system of the jaw exoskeleton concept by Evans et al. (2016). (Top left) The drive mechanism redirects the torque via a gear system from the vertically placed DC motor over the bracket to the mouthpiece. Only vertical assistance can be provided. The bracket is rigidly attached to the gear system and includes a counterweight on the back to balance the system and reduce the load on the actuator. ^©^2016 IEEE. Reprinted with permission. (Top right) The mouthpiece consists of a linear slide and a strain gauge placed on the slide to measure the forces acting on the mouthpiece. A chin strap enables transmitting forces to the mandible in both vertical directions. The mandible fitting connects to the lower teeth and can be replaced. ^©^2016 IEEE. Reprinted with permission. (Bottom) The control system design features a hierarchical structure with a motor controller, an impedance controller, and a trajectory planner. The motor controller is responsible for the actuation, the impedance controller for the active vertical compliance, and the trajectory planner for the trajectory following tasks. The system can be cut off from power through a hand-held safety button. Adapted from Evans et al. (2016).

The mouthpiece is connected through a linear slide, compensating passively for the mandible’s translational movements. A head brace fixes the mounted system in place. Actuating the rehabilitation system is a DC motor capable of providing a maximum torque of 0.5 N m, resulting in approximately 13 N around the mouthpiece. To save space the actuator is placed vertically and torques are redirected by gear systems (Figure 6).

The motion control strategy is based on a hierarchical approach with a motor controller at the lowest level, an impedance controller providing active vertical compliance at the mid-level, and a trajectory planner at the highest level (Figure 6). A hand-held safety button must be continuously pressed to keep the device operational. Sensory information is acquired by a strain gauge placed on the linear slide and a motor encoder (Figure 6). EMG data is recorded to assess the training performance but deemed to be too noisy to be used for control. Besides progressive stretching and resistance training, the device was intended to involve the patient actively in the training process and encourage thus the motor learning process by implementing assist-as-needed routines and providing visual feedback in trajectory following tasks. Data should be collected to evaluate the training performance and characterize the patient’s particular TMD.

However, the device lacks a biomechanical model as a development basis and validation in a simulation environment. Since the device was not realized as a physical prototype and was meant as a design concept, no clinical studies were conducted.

#### 5.3.3 An assistive device for strengthening oral motor function

**FIGURE 7:**
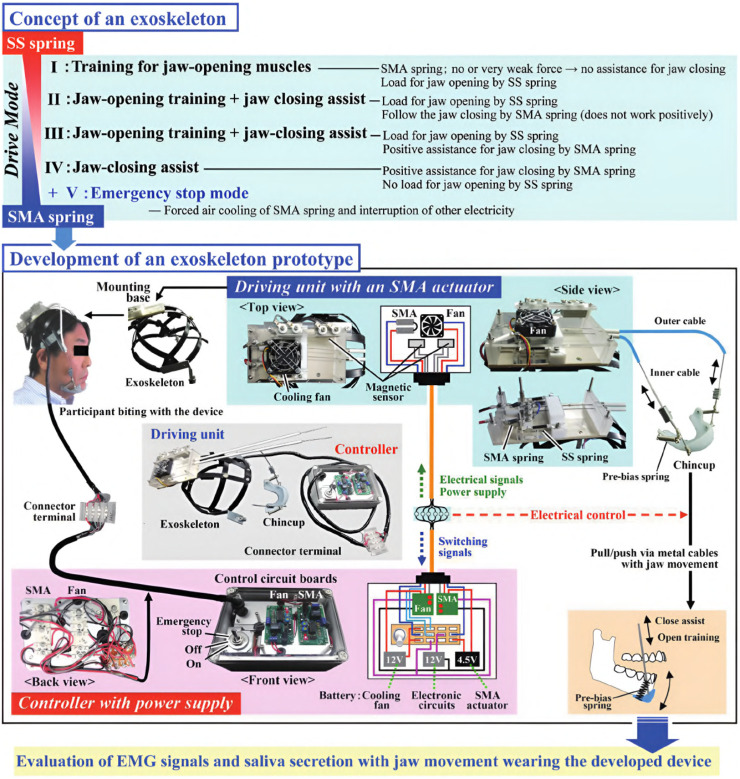
An overview of the exoskeleton by Kameda et al. (2021). The device implements four drive modes. In the first mode, the active part of the device is disabled, only the passive stainless steel (SS) springs act against mouth opening. In the second mode, the SMA springs are additionally heated, providing a force to follow the closing trajectory. The third mode is similar to the second but with a higher force output, actively assisting the closing motion. In the fourth mode, the device only provides assistance in the closing direction and no resistance during the opening movement. The exoskeleton itself comprises a chin cup attached to a head mount by cables and SMA and passive springs. Only forces in the closing direction can be applied. ^©^2021 The Japanese Society for Dental Materials and Devices. Reprinted with permission.

**FIGURE 8:**
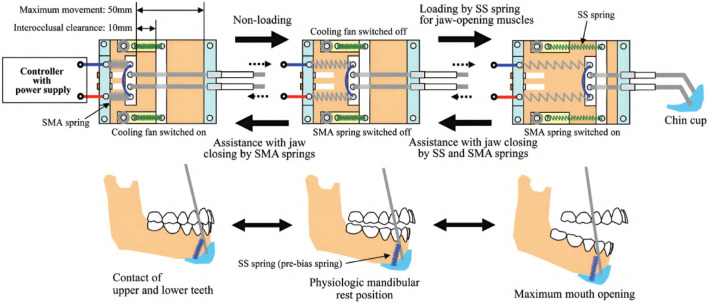
An illustration of the drive mechanism of the jaw exoskeleton by Kameda et al. (2021). The main actuator system is based on SMA springs, which contract when heated by an electric current. The springs expand again when cooled down. A fan is triggered by a magnetic sensor to accelerate the cooling process. Stainless steel (SS) springs are added to counteract the time delay of the SMA springs. The cup is attached to a head mount and the actuator system by cables. ^©^2021 The Japanese Society for Dental Materials and Devices. Reprinted with permission.

The research objective of Kameda et al. was to develop an assistive device for strengthening oral motor function by increasing muscular activity during jaw opening and closing (Kameda et al., 2021). By preventing oral frailty, which leads to potentially decreased occlusal force, physical hypofunction, and malnutrition, the life expectancy of the elderly should be prolonged, and a healthy social life should be facilitated. Based on a chin cup attached to a head mount by cables, the device is equipped with shape-memory alloy (SMA) springs characterized by low power consumption to provide a maximum force of 3 N. While the exoskeleton is compliant in every direction due to the flexible cable system and springs, the chin cup is attached by form closure. Thus, assistance can be only provided while closing the mouth. Still, resistance can be applied during jaw opening. In an inactive state, the mouth can be opened, working against the passive forces of the springs. As soon as the SMA springs are heated by applying a current, a closing force is created. A fan helps cool down the springs faster afterward, activated and deactivated by a magnetic sensor switching system, which must be adjusted to each user’s anatomical dimensions to match the timing of exerted forces. Regular passive springs are additionally integrated into the system to counteract the time delay induced by the duration it takes to heat the SMA springs. In total, the device weighs about 700 g. An overview of the various driving modes, the device itself, and the drive mechanism is provided in Figures 7, 8.

Similar to the approach by Evans et al., neither were models created to design the system nor was the device validated in a simulation environment. However, a user study was conducted to evaluate the device’s effectiveness and determine if saliva secretion is stimulated. Ten healthy male participants with an average age of 55 years were asked to wear the device and perform a series of jaw opening and closing movements while simultaneously measuring EMG signals and saliva secretion. The recorded data was processed and compared to a control group without the exoskeleton, showing saliva secretion helpful in maintaining good oral hygiene was stimulated, and an opening load and closing assistance increase of 25% and 15% could be achieved in terms of muscle activity, respectively.

#### 5.3.4 A soft exoskeleton approach for rehabilitating temporomandibular disorders

**FIGURE 9:**
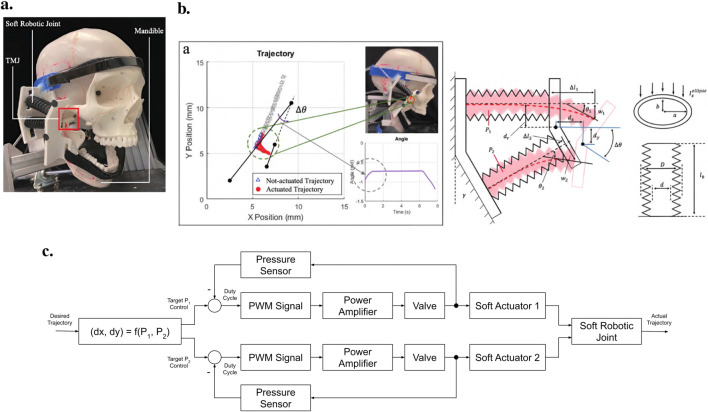
An overview of the jaw exoskeleton design and testing by Zhang et al. (2021). **(a)** The device consists of two pneumatic joints attached to the head and chin by an adjustable fixing ribbon. The joints are capable of providing assistance in two DoF. ^©^2021 IEEE. Reprinted with permission. **(b)** The exoskeleton was evaluated on a physical skull model, which only included the rigid bones. The mandible was actuated and the exoskeleton only provided assistance in the sagittal plane. The image shows the trajectory of two markers placed on the end-effector of the pneumatic joints. The two black lines represent the orientation of the end-effector at two distinct time points. The triangles indicate the non-actuated position of the end-effector, and the red dots indicate the actuated position. By pushing the condyles out of the fossae, a translational movement is generated leading to a wider opening of the mouth. The 2D model of the pneumatic joints shows the bellow-shaped cylinders attached to a base and an end-effector. ^©^2021 IEEE. Reprinted with permission. **(c)** The control scheme utilizes a feed-forward approach, where pressure signals are generated using an inverse kinematic model of the pneumatic joints to track a predefined trajectory. Pressure is monitored via sensors and regulated through control valves. Adapted from Zhang et al. (2021).

The first exoskeleton design to use a soft approach introducing compliance in every direction was presented by Zhang et al., in 2021 (Zhang et al., 2021). Actuated by two pneumatic joints, the device was attached to the head and chin by an adjustable fixing ribbon, resulting in 340 g total weight (Figure 9a). One such pneumatic joint comprises two bellows-shaped cylinders attached to a 3D-printed base on one end and an end-effector similar in geometry to the base on the other (Figure 9b). Consequently, neither cylinder can move independently. However, each cylinder can be pressurized individually, leading to a two DoF system.

The control scheme is based on a feed-forward approach, where the pressure signals are generated by an inverse kinematic model of the pneumatic joints to follow a predefined trajectory (Figure 9c). It remains unclear if and how the pressure is controlled in a feedback loop. Except for pressure sensors, no further sensory systems were integrated. The capability of the pneumatic joints to reproduce mandibular motion in the sagittal plane was evaluated by calculating a trajectory and comparing it to the actual trajectory of the actuators by tracking the movement of two markers placed on an actuator. A deviation of less than 1 mm was achieved, with little oscillations of the end-effector. However, it was not mentioned that the calculated reference trajectories correspond to actual jaw movements. In return, to validate its ability to assist with jaw movement, the proposed exoskeleton was tested on a physical skull model, which only included the rigid bones (Figure 9b). Assuming that the patient generates the main forces and motions, the model’s mandible was connected to an actuator system. As a result, the authors stated that the device could reproduce the mandible’s trajectory but that the sufficiency of the provided assistance for training needs further evaluation. Again, no biomechanical model was created to design the system, and no clinical studies were conducted. In summary, the research seems to focus more on the actuator system than the rehabilitation device itself.

Table 1 compares the presented approaches, summarizing the state-of-the-art research on rehabilitative exoskeletons for TMDs. The table includes key aspects such as design characteristics, actuation, power transmission, force/torque, weight, DoF, control strategies, human-machine interaction, adaptability, biomechanical models, and validation and evaluation of each device.

**TABLE 1:** This table provides a comparison of state-of-the-art rehabilitative exoskeletons developed for treating TMDs.

	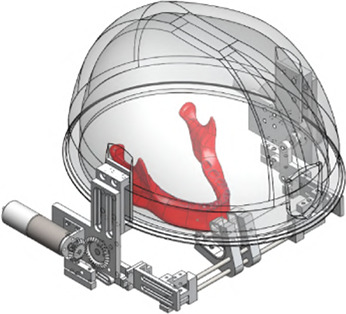 Wang et al.	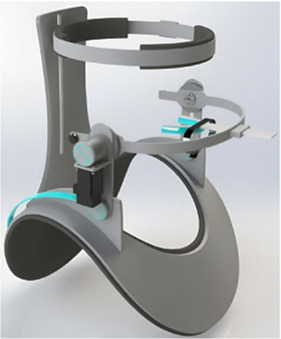 Evans et al.	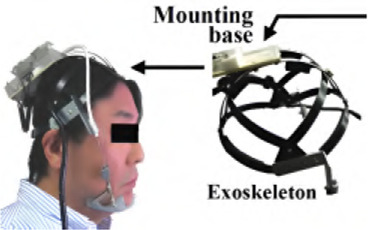 Kameda et al.	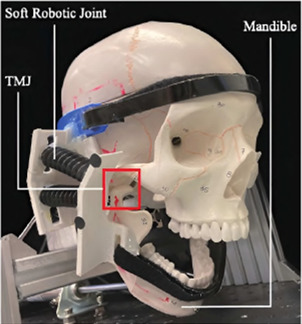 Zhang et al.
References	Wang et al. (2010), Wang et al. (2014); Wang (2014)	Evans et al. (2016)	Kameda et al. (2021)	Zhang et al. (2021)
Year	2010–2014	2016	2021	2021
Design Characteristics	Helmet + 4-bar linkage	Shoulder-mounted bracket	Chin cup attached to head mount by cables	Compliant pneumatic head-mounted joints
Main Materials	Aluminium	Aluminium + pvc	Resin + steel	Plastics + fabric
Actuation	DC motor	DC motor	SMA springs	pneumatic
Power Transmission	Rigid (chin holder)	Rigid (mouthpiece + chin strap)	Cables (+ chin cup)	Compliant (chin strap)
Force/Torque	10 N–30 N	0.5 N m (13 N)	3 N	-
Weight	( < 1,000 g)	-	700 g	340 g
DoF (Assistance)	1 (up/down)	2 (up/down)	1 (up)	2 (up/down)
Control Strategy	Feedback PID (position)	Hierarchical (position)	On/off (SMA)	Feed-forward (pressure)
Human-Machine Interaction	force, angle, Hall sensors	GUI, force, position sensors, safety button	EMG (data recording)	-
Adaptability	Yes	Yes	Yes	Yes
Biomechanical Model	Rigid jaw model	No	No	No
Validation sim./phys.	Jaw + device/jaw + prototype	No/no	No/no	Device/jaw + prototype
Studies	No	No	10 subjects	No

The force/torque values refer to the maximum output of the actuation system at the jaw attachment point. The DoF row relates to the degrees of freedom of the device itself, and the parentheses indicate the possible directions of active assistance. The control strategy row describes the implemented control concept with the control target in parentheses. The human-machine interaction is characterized by the sensory systems used to monitor the patient’s movements and the device’s state. The adaptability row indicates whether the device can be adjusted for different patients. The biomechanical model row refers to the presence of a model of the human jaw in the development process. Validation sim./phys. denotes whether the device was validated in a simulation or physical environment. Simulation and physical validation are separated by a slash. Medical studies were only conducted by Kameda et al., involving 10 subjects. Limitations in validation and studies highlight the early stage of development in this field. The weight listed for the device by Wang et al. is a specified design requirement rather than a measured value (denoted in parentheses). ^©^Copyright notices are included in Figures 3, 5, 7, 9.

## 6 Discussion

Analyzing the presented approaches, it becomes evident that the current state of the art in powered rehabilitative exoskeletons for TMDs is still in an early stage of development. The following discussion aims to highlight the opportunities, limitations, and practical challenges of these devices, providing insights into their potential clinical applications to assist in the rehabilitation of TMDs, ethical considerations, and future research directions. The structure of the discussion is based on the identified requirements and scientific challenges in Subsection 5.2 and evaluating and comparing the reviewed designs and methodologies against these criteria.

### 6.1 User safety

The development of jaw exoskeletons for rehabilitative purposes has progressed from rigid, mechanical designs to softer, more compliant, and hybrid approaches. The progression toward soft and hybrid designs highlights an increasing emphasis on patient safety, as these approaches inherently reduce the risk of injury during physical interaction.

Ensuring user safety during physical interaction with exoskeletons is critical, particularly when applying forces to the mandible. The unique anatomy of the masticatory system necessitates careful consideration to avoid excessive or improperly directed forces that could damage the temporomandibular joint or dental structures. Of the reviewed devices, only the approach by Kameda et al. was tested on humans, demonstrating reliable power transmission, though limited to the closing direction via the form closure of the chin cup with the lower jaw. Zhang et al.’s pneumatic design relies on adhesion to the mandible for force transfer in tests with a physical skull model, raising concerns about the safety and stability of the device during active patient use. Insufficient information is provided about the reliability of power transmission and safety mechanisms in the devices by Wang et al. and Evans et al., leaving uncertainties about potential risks. While Evans et al.‘s device supposedly secures a stable connection to the mandible via a mouthpiece and chin strap, Wang et al.’s device attaches superficially to the lower jaw at four points, potentially compromising stability during active training due to factors such as soft skin tissue or patient movement.

The devices by Kameda et al. and Zhang et al. offer good kinematic compatibility with the mandible, as their soft designs inherently adapt to the natural motions of the masticatory system. Conversely, the more rigid designs by Wang et al. and Evans et al. may restrict the complex combination of rotational and translational movements of the mandible, potentially causing discomfort or injury if the device fails to follow the patient’s jaw motion accurately. This concern should be addressed in future studies. However, the low forces and torques applicable in all devices at this stage of development reduce the immediate risk of injury.

None of the reviewed devices adequately address the challenge of limiting forces to safe thresholds across all jaw positions and movement patterns. The implemented control concepts generally lack the sophistication to adapt to changing biomechanical parameters or environmental conditions, with only Evans et al.‘s never-implemented hierarchical approach showing potential for adaptive capability.

A significant gap across all designs is the absence of comprehensive fail-safe mechanisms that incorporate redundant and multimodal sensor systems to address sensor or actuator failure. Although Evans et al. proposed a safety button to cut off power to the device, it remains unclear how the system would respond to unexpected resistance or pain signals from the patient. Future designs must integrate dynamic safety systems capable of adapting to patient-specific anatomical variations and responding to unexpected resistance or pain signals. Additionally, they should implement redundant emergency protocols to handle component failures effectively.

### 6.2 Robust human-robot cognitive interaction

Human-machine interaction varies considerably across devices, with significant limitations in their cognitive interaction capabilities. Wang et al. and Evans et al. incorporated multiple sensors, including force, angle, and Hall sensors, as well as EMG data recording, to monitor device performance and patient activity. Evans et al. envisioned using visual feedback and assist-as-needed routines to actively engage patients during training. However, these features remain conceptual, and their effectiveness has not been validated. Kameda et al. relied solely on EMG sensors to monitor muscle activity and evaluate device effectiveness, while Zhang et al. did not specify any additional sensory systems beyond pressure sensors for pneumatic control.

The integration of multimodal sensory systems remains a significant challenge. Sensors like EMG, motion trackers, and force sensors are often influenced by noise, patient variability, and placement inconsistencies, which may compromise their reliability. While Kameda et al. and Evans et al. use EMG sensors to evaluate training performance, the data is not utilized for control purposes or user intention detection in any of the designs. Moreover, none of the approaches adequately describe methods for detecting and interpreting user intentions, a fundamental requirement for implementing assist-as-needed training routines or promoting active patient participation.

Control strategies differ significantly across the devices, reflecting varying levels of sophistication. Wang et al. employed feedback-based PID controllers for position control, which are simple and effective but lack adaptability to changing biomechanical parameters. Zhang et al. used a feed-forward approach to generate pressure signals for pneumatic joints, which assumes pre-defined trajectories and does not account for real-time patient variability, although inherently given through the soft design. Kameda et al. implemented a basic on/off control for SMA-driven assistance, limiting its flexibility during rehabilitation.

Evans et al. proposed the most sophisticated control strategy, featuring a hierarchical framework with a low-level motor controller, mid-level impedance controller, and high-level trajectory planner. This structure is well-suited for implementing assist-as-needed routines, as the introduced impedance can be used to simulate different levels of passive resistance or compliance based on patient performance or preferences. The concept, however, was never realized in practice. The lack of adaptive or learning-based control strategies, such as machine learning algorithms, is a notable gap in all devices. Machine learning techniques could enable adaptive control by analyzing patient-specific data in real-time, optimizing training routines, and detecting user intentions more accurately.

### 6.3 Forces and range of motion

The actuation systems used in jaw exoskeletons reflect diverse design priorities with significant implications for performance, safety, and usability. Wang et al. and Evans et al. employed DC motors, which offer high precision and are well-suited for trajectory tracking. However, DC motors are typically heavier and bulkier, which can limit the wearability and portability of these devices. Kameda et al. utilized SMA springs, which are lightweight and compliant but suffer from slow actuation speeds and limited force output. In contrast, Zhang et al. introduced pneumatic joints, which provide inherent compliance and a high power-to-weight ratio. However, pneumatic systems often require external compressors, adding complexity and reducing portability.

All approaches meet the requirements for the vertical range of motion, but other directions are not actively covered in any of the designs. Regarding force output, only the device by Wang et al. can provide the minimum opening force of 15 N–25 N established in the literature, with limited information about maximum applicable forces across all designs. This suggests that further research is necessary to map the complete RoM and mandibular forces needed for effective rehabilitation.

In terms of DoFs, the systems range from one to two across the devices. Wang et al. and Zhang et al. implemented systems capable of assisting both opening and closing jaw motions, making them more versatile for rehabilitation exercises. In contrast, Kameda et al. focused solely on providing assistance in the closing direction, with resistance during opening achieved passively. Evans et al. proposed a concept with two DoFs, but its functionality remains theoretical, as no physical or virtual prototype was developed.

While the inclusion of multiple DoFs enhances the device’s ability to replicate natural jaw movements, none of the systems fully capture the six DoFs required for comprehensive jaw rehabilitation. This limitation restricts their applicability to more complex jaw motions, such as lateral excursions or combined rotational and translational movements. How many DoFs are actually necessary for effective rehabilitation, however, remains an open question, as the optimal number likely depends on the specific training goals and patient needs.

### 6.4 Wearability and portability

Wearability and portability represent critical factors influencing patient acceptance and treatment adherence. Since the weights of the exoskeletons are all under 1 kg, the basic wearability requirements in this regard are met. However, the reviewed devices demonstrate varying approaches to these challenges, with each making different compromises.

Rigid exoskeletons like those developed by Wang et al. offer stable support but significantly impact aesthetics and comfort. Their external mounting systems create visible alterations to appearance that may increase self-consciousness in social settings. Zhang et al.’s soft pneumatic approach improves wearability through its flexible, lightweight design but requires attachment to external pneumatic infrastructure, substantially limiting mobility during therapy sessions.

Kameda et al.’s SMA-based solution achieves better portability but with compromises in actuation speed and force capabilities that may limit therapeutic efficacy. Kameda et al. also noted that their battery power supply needs further improvement due to rapid drainage, highlighting power supply as a potential limiting factor that should be considered early in the development process.

None of the current designs successfully balance all aspects of wearability: aesthetic acceptability, comfort during extended use, and sufficient unobtrusiveness to allow for normal daily activities if the device is also intended for patients with accident injuries or joint prosthetics. Such patients may be limited in possible movements and activities, making continuous wear of the device necessary.

Since the actuators in Wang et al., Evans et al., and Kameda et al.‘s approaches are attached directly to the head-mounted components, potential improvements could be achieved by relocating the actuators to the hip or back, thereby reducing the effective weight on the head.

### 6.5 Flexibility and data collection

Adaptability to individual patients is facilitated to some extent in all devices. Wang et al. introduced adjustable linkage lengths, Evans et al. used replaceable mouthpieces, and Zhang et al. employed adjustable fixing ribbons. Kameda et al.’s device allows for spring adjustments, but this process is intricate and time-consuming. Despite these adaptability features, none of the devices explicitly address customization for different types of TMDs or patient-specific anatomical variations.

For effective rehabilitation, jaw exoskeletons must accommodate the specific needs of different TMD presentations. For example, patients with disc displacement might require different movement patterns and force profiles compared to those with myofascial pain or arthritis. Current devices lack the customization capabilities necessary to address this clinical diversity, limiting their therapeutic potential. Although a device for all forms of TMD seems unreasonable, the ability to adjust training parameters based on patient-specific needs would significantly enhance rehabilitation outcomes.

The potential for collecting data to objectify the diagnosis and prognosis of TMDs or track training progress is only elaborated on by Evans et al. and implemented by Kameda et al. during their user study. Evans et al. proposed splitting function-relevant computations and data acquisition, dedicating multiple onboard processors to different tasks. However, none of the approaches provide detailed information about the concrete technical implementation of their proposed training tasks and data collection, leaving questions about the construction of assist-as-needed routines and data-driven progress tracking largely unanswered.

Additionally, the absence of progressive resistance and guided motion patterns tailored to recovery stages represents a significant gap. Rehabilitation typically requires gradually increasing challenges as recovery progresses, yet current devices offer limited adaptability or information to this therapeutic requirement. The integration of assessment tools to monitor progress and automatically adjust therapy parameters would significantly enhance rehabilitation efficacy but remains underdeveloped and underexplored in existing systems.

### 6.6 Neurological and neuromuscular rehabilitation

None of the reviewed devices adequately addresses the requirements for effective neurological rehabilitation of the masticatory system. While Evans et al. proposed implementing assist-as-needed training routines and game-like tasks to encourage active participation, these concepts were never realized. The other approaches mention various training routines but provide no details about their concrete technical implementation.

Effective neurological rehabilitation to engage the patient in the rehabilitation process requires providing appropriate feedback and progressively challenging tasks across the entire range of possible movements. However, the reviewed devices lack sophisticated feedback mechanisms such as visual guidance systems or performance metrics that could enhance motor learning processes. The absence of game-like interfaces or other engagement strategies further limits their potential for promoting active patient participation.

Evans et al.’s concept comes closest to addressing these needs with its proposed visual feedback system and hierarchical control structure suitable for implementing assist-as-needed support. However, without practical implementation and validation, its effectiveness remains theoretical. The lack of attention to neurological rehabilitation aspects across all designs represents a significant missed opportunity, particularly considering the potential benefits for patients with neuromuscular components to their TMD.

Future jaw exoskeletons should incorporate interactive visual feedback systems, progressive challenge adjustment based on performance metrics, and engaging training paradigms to optimize neurological rehabilitation outcomes. Such features would not only enhance treatment efficacy but could also improve patient motivation and adherence to rehabilitation protocols.

### 6.7 Usability and acceptance

Patient comfort and usability are critical factors that remain underexplored across all designs. The lack of clinical studies means that no statements about the usability and acceptance of the devices from a user’s perspective can be made with certainty. However, one might reasonably assume that acceptance of soft approaches like Zhang et al.’s would be higher due to greater comfort, ergonomics, and the inherent compliance that leads to more natural behavior.

Usability extends beyond physical comfort, including ease of donning and doffing the device, intuitiveness of controls, and minimal disruption to speech and other oral functions. Current designs generally appear easily mountable on the head or shoulders but lack intuitive user interfaces with sophisticated control concepts, potentially impeding usability. Moreover, only the exoskeleton concept by Evans et al. integrates a system that gives the user full control over the system–specifically, the hand-held safety button that must be continuously pressed during operation.

User acceptance is further complicated by the intimate nature of oral devices, where even minor discomfort can lead to rejection. Kameda et al.‘s study provided some insight into muscle activations and saliva production during device use, but gave no indication of patient comfort or acceptance. The lack of longitudinal user studies across all designs raises questions about long-term acceptance and compliance, particularly when prolonged treatment periods are required for TMD rehabilitation.

Future designs must prioritize user-centered development approaches incorporating patient feedback throughout the design process, focusing on comfort, aesthetics, and intuitive operation to maximize acceptance and treatment adherence.

### 6.8 Biomechanical model and adaptive control

The lack of comprehensive biomechanical models to design and validate these systems in simulation environments represents one of the most significant deficiencies across all approaches. The only model created–by Wang et al. – was not validated with proper data and was limited in detail. As a result, rapid prototyping, considering patient safety and comfort early on in the design process, was likely compromised. The absence of detailed models also limits the ability to simulate different TMD presentations and patient-specific anatomical variations, which are critical for developing adaptable and personalized rehabilitation strategies. Furthermore, model-based controllers, which could potentially enhance control accuracy and adaptation, were not employed in any of the designs.

Developing appropriate biomechanical models involves determining the necessary level of detail and complexity based on the intended use–whether for design validation, comparison between healthy individuals and TMD patients, or device control. The absence of such models in the development process is likely limiting the sophistication and effectiveness of the resulting exoskeletons.

Without detailed biomechanical understanding integrated into the design process, the devices may fail to accurately reproduce natural jaw movements or apply forces in optimal directions and magnitudes. Additionally, developing adaptive control strategies that adjust to individual differences and real-time changes in biomechanical parameters becomes significantly more challenging without model-based foundations.

Future research must prioritize the development of validated biomechanical models that can guide exoskeleton design, inform control strategies, and enable meaningful simulation-based testing before physical prototyping. Such models should incorporate rigid body dynamics for overall movement patterns and finite element approaches for detailed stress and strain analysis of critical components like the TMJ discs and associated tissues.

### 6.9 Practical applications in TMD rehabilitation

The reviewed jaw exoskeletons, despite their limitations, demonstrate potential for practical application in TMD rehabilitation by addressing several key therapeutic needs. Since all devices provide assistance in the vertical direction, they might align well with fundamental physiotherapeutic requirements for treating myogenous and non-degenerative arthrogenous TMDs as outlined in Section 3. For example, the devices could support progressive stretching exercises, resistance training, and post-surgical rehabilitation, essential components of multimodal TMD treatment strategies.

Vertical assistance capabilities make these devices particularly suitable for implementing progressive stretching exercises, which have been shown to reverse degenerative changes, alleviate pain, and mitigate motion limitations associated with arthrogenous TMDs, notably disc displacements without reduction (Armijo-Olivo et al., 2016; Wänman et al., 2016). This functionality directly addresses the physiotherapeutic principle that regular movement maintains the health of synovial joints like the TMJ (Kraaijenga et al., 2014; Israel and Syrop, 1997).

Resistance training functionality, present in varying degrees across the devices, offers potential for strengthening masticatory muscles–a critical component in addressing myalgia, which represents approximately 80% of TMD cases (Durham et al., 2015; List and Jensen, 2017). Wang et al.’s and Evans et al.’s devices particularly support this application through their bidirectional force capabilities, potentially enhancing muscular activity during both opening and closing movements similar to what Kameda et al. demonstrated in their user study.

For post-surgical rehabilitation scenarios, where physiotherapeutic measures serve as valuable treatment adjuncts (Abboud et al., 2018; du Plessis et al., 2021), these devices could provide controlled, guided motion to prevent stiffness while respecting surgical recovery constraints. The passive compliance featured in Zhang et al.’s pneumatic approach might be particularly beneficial in these sensitive applications where gentler assistance is required.

The integrated sensors in these devices–particularly evident in Wang et al.’s and Evans et al.‘s approaches–facilitate objective data collection that addresses the subjective nature of conventional rehabilitation assessment identified in Subsection 5.2.5. These monitoring capabilities could transform TMD treatment by enabling:

1. Quantifiable progress tracking through consistent measurement of RoM, force generation, and movement patterns
2. Enhanced treatment personalization based on objective biomechanical data
3. Earlier detection of compensation patterns that might otherwise lead to secondary issues
4. Remote monitoring capabilities, allowing clinicians to assess patient progress between appointments

For patients with complex TMD manifestations involving neuromuscular components, Evans et al.’s proposed visual feedback system holds particular promise for neurological rehabilitation through motor relearning principles. This application directly addresses the potential benefit of enhanced neurological rehabilitation outlined in Subsection 5.2.6.

However, translating these theoretical capabilities into clinical practice remains challenging. No device currently combines all necessary elements–robust vertical assistance, appropriate force generation, comprehensive sensing, and user-friendly interfaces–required for comprehensive TMD rehabilitation. Moreover, the absence of clinical validation studies (except for Kameda et al.’s investigation) leaves important questions unanswered about real-world therapeutic efficacy and patient acceptance.

Future development should prioritize devices that can smoothly transition between multiple rehabilitation modalities–passive stretching, active resistance, and neuromuscular reeducation–while incorporating appropriate feedback mechanisms. Establishing standardized protocols for different TMD presentations would further enhance the practical utility of these devices as adjuncts to conventional physiotherapy or as a home-based continuation of clinical interventions.

### 6.10 Ethical considerations

Jaw exoskeleton development and deployment introduce ethical considerations beyond technical aspects. Nasr et al. emphasize the importance of establishing clear liability frameworks for cases involving device malfunction, where responsibilities between manufacturers, clinicians, and patients may be ambiguous. Transparent communication with users about device capabilities, functionality, limitations, and risks is essential for informed consent and building trust (Nasr et al., 2024).

Respecting patient autonomy requires robust informed consent processes, particularly given these devices’ experimental nature at this stage. Tu and Gao advocate for communicating study information in simple language, supplemented with videos and demonstrations to enhance understanding. The consent process may need adaptation based on education levels, cognitive abilities, and clinical conditions. Complete disclosure of limitations, risks, and current evidence is fundamental to maintaining ethical standards (Tu and Gao, 2021).

Devices collecting movement or other biometric data raise privacy concerns, as jaw motions can reveal sensitive information about eating patterns, speech, and emotional states. Canali et al. note that the literature often separates technical and ethical discussions. The relationship between specific technical implementations and ethical considerations remains understudied (Canali et al., 2022). Thus, ethical considerations such as data privacy, patient comfort, and safety must be linked to technical design choices.

Equitable access represents another critical ethical dimension. Advanced robotic systems’ high cost and complexity may limit their accessibility, particularly for patients in low-resource settings. Ethical considerations must address this potential to exacerbate healthcare inequalities, ensuring that technological advancements benefit diverse patient populations. Future designs should strive for cost-effective solutions without compromising functionality or safety. For evaluating the devices in the developing process itself, Tu and Gao emphasize that including diverse participants is essential for minimizing selection bias and ensuring the generalizability of results (Tu and Gao, 2021).

Ethical management of therapeutic expectations requires avoiding overstatement of potential benefits during recruitment or deployment. Transparent communication about these devices’ experimental status helps prevent the exploitation of vulnerable patients seeking relief from debilitating TMD symptoms.

The authors of the reviewed studies have not explicitly addressed ethical considerations in their work, focusing primarily on technical aspects. However, the multifaceted nature of jaw exoskeleton development and deployment necessitates a broader ethical perspective to ensure patient safety, privacy, accessibility, and informed consent. Future research should integrate ethical considerations into the design process, emphasizing patient-centered care and equitable access to rehabilitative technologies.

### 6.11 Future directions

The current state of jaw exoskeletons represents an early stage of development, with significant opportunities for advancement. Future research should prioritize several key areas to advance the field toward clinical viability.

Developing high-fidelity biomechanical models is essential for guiding device design and control strategies. These models must account for jaw mechanics’ complex, patient-specific nature and incorporate data from imaging and motion capture to create personalized rehabilitation approaches. Such models would enable a more accurate simulation of intervention effects before physical implementation, potentially accelerating development while improving safety.

Advanced sensing and actuation technologies should be explored to overcome current limitations in force output, degrees of freedom, and adaptability. Hybrid actuation systems combining the precision of motors with the compliance of soft materials could offer promising solutions for balancing performance requirements with safety considerations. Integrating embedded sensing within soft structures could reduce bulk while improving motion detection accuracy.

Machine learning algorithms offer significant potential for personalizing rehabilitation protocols based on patient progress and movement patterns. These approaches could enable adaptive control that adjusts assistance levels in real-time based on patient effort and performance metrics. Implementing such systems would require careful validation to ensure they respond appropriately to the diverse presentation patterns of TMD. However, additional fail-safe and safety mechanisms must be integrated since current machine learning algorithms are still often considered black boxes that may not always be predictable.

Miniaturization and aesthetic improvement represent crucial directions for enhancing wearability and acceptance. Future designs should strive to minimize visible components while maintaining functionality, potentially through innovations in materials science and compact actuation technologies. Developing softer, visually appealing, and ergonomic designs could significantly enhance patient comfort and adherence to treatment protocols.

Rigorous clinical validation through controlled trials comparing jaw exoskeletons to conventional therapy approaches is essential for establishing their therapeutic value. These studies should assess not only biomechanical outcomes but also pain reduction, quality of life improvements, and functional gains in daily activities. Long-term follow-up is necessary to determine whether improvements persist after device use concludes.

Interdisciplinary collaboration between engineers, clinicians, patients, and ethicists will be critical for addressing the multifaceted challenges of jaw exoskeleton development. Such collaboration ensures that technical innovations align with clinical needs and ethical considerations, potentially accelerating translation to practice while maintaining patient-centered design principles.

Cost-effective manufacturing approaches should be explored to improve accessibility, particularly in resource-limited settings. Innovations in materials and production methods could reduce costs without compromising essential functionality, potentially expanding the reach of these rehabilitative technologies to broader patient populations.

Jaw exoskeletons hold significant potential as supplements to conventional physiotherapy for TMDs, offering personalized, quantifiable, and potentially more engaging rehabilitation possibilities. However, realizing this potential will require addressing the substantial challenges identified in this review through coordinated, patient-centered research efforts. Moreover, given the multifaceted nature of TMDs and individual patient differences, developing a single solution for every case remains difficult. Therefore, a multimodal approach that combines exoskeletons with other treatment methods likely offers the most promising path forward. Initial diagnosis and ongoing progress assessment should remain under expert clinical supervision, with the exoskeleton serving as an additional tool to support therapists and enhance treatment convenience for patients.

The current literature on jaw exoskeletons provides valuable technical insights and conceptual frameworks, albeit neglecting comprehensive clinical validation and ethical considerations. Consequently, as the development of such devices is still in an early stage, many open questions remain to be answered before they can be effectively implemented in clinical practice. The comprehensive scientific foundation to build upon needs still to be created.

## Abbreviations

DoF, degrees of freedom; EEG, electroencephalography; EMG, electromyography; IMU, inertial measurement unit; RoM, range of motion; SMA, shape-memory alloy; TMD, temporomandibular disorder; TMJ, temporomandibular joint.
